# Supplementary material for: Navigating Cultural Challenges in Transplant Surgery: Insights from Turkish Surgeons
Source: Healthcare (Basel). 2024 Jun 24;12(13):1252. doi: 10.3390/healthcare12131252 (PMC11240981; doi:10.3390/healthcare12131252)
Supplement: Supplementary file 1 [file healthcare-12-01252-s001.zip › healthcare-3056308-supplementary.pdf]

Table_1_Characteristics of the transplant surgeons

No	Pseudonym	Gender	Age	Marital Status	Parenting
001	Ayhan	M	66	Married	Y
002	Saniye	F	45	Married	Y
003	Şeref	M	45	Married	Y
004	Arda	M	57	Widowed	Y
005	Oskay	M	63	Married	Y
006	Ahmet	M	69	Married	Y
007	Fatih	M	57	Married	Y
008	Ulvi	M	43	Married	Y
009	Hatip	M	58	Married	Y
010	Yusuf	M	45	Married	Y
011	Ali	M	55	Married	Y
012	Aynur	F	44	Married	Y
013	Toygar	M	45	Married	Y
014	Burhan	M	39	Married	Y
015	Cihat	M	45	Married	Y
016	Emir	M	39	Married	Y
017	Bahriye	F	45	Married	Y
018	Emre	M	37	Married	Y
019	Serhat	M	46	Married	Y
020	Sergen	M	60	Married	Y
021	Emre	M	44	Married	Y
